# Distinct neural responses of morningness and eveningness chronotype to homeostatic sleep pressure revealed by resting‐state functional magnetic resonance imaging

**DOI:** 10.1111/cns.13887

**Published:** 2022-06-14

**Authors:** Haien Wang, Yun Tian, Yulin Wang, Qinghua He, Jiang Qiu, Tingyong Feng, Hong Chen, Xu Lei

**Affiliations:** ^1^ Sleep and NeuroImaging Center, Faculty of Psychology Southwest University Chongqing China; ^2^ Key Laboratory of Cognition and Personality (Southwest University), Ministry of Education Chongqing China

**Keywords:** angular, chronotype, homeostatic sleep pressure, insular, rsfMRI

## Abstract

**Background:**

Chronotype is an appropriate variable to investigate sleep homeostatic and circadian rhythm. Based on functional MRI, the resting‐state functional connectivity (rsFC) of insula‐angular decrease with the increase in homeostatic sleep pressure (HSP). However, the distinct neural response of different chronotype remained to be clarified. Therefore, we investigated how HSP influenced insular‐angular neural interaction of different chronotype.

**Methods:**

64 morningness‐chronotype (MCPs) and 128 eveningness‐chronotype participants (ECPs) received resting‐state functional MRI (rsfMRI) scan. HSP was divided into three levels (Low, Medium, and High) based on the elapsed time awake. Insular‐angular rsFC was calculated for MCPs and ECPs on each HSP.

**Results:**

As the levels of HSP increased, the negative rsFC between right insular and bilateral angular increased in MCPs while decreased in ECPs. Specifically, ECPs compared with MCPs showed lower rsFC at medium levels of HSP, but higher rsFC at high levels of HSP. In addition, ECPs compared with MCPs exhibited lower rsFC between right insular and right angular at low levels of HSP.

**Conclusion:**

The distinct modes of rsFC was found in different chronotype in response to HSP. The results provided the foundation and evidence for investigating the processes of circadian rhythm and sleep homeostatic.

## Graphical abstract

The distinct modes of rsFC was found in different chronotype in response to HSP. As the levels of HSP increased, the negative rsFC between right insular and bilateral angular increased in MCPs while decreased in ECPs. Specifically, ECPs compared with MCPs showed lower rsFC at medium levels of HSP, but higher rsFC at high levels of HSP. In addition, ECPs compared with MCPs exhibited lower rsFC between right insular and right angular at low levels of HSP. The results provided the foundation and evidence for investigating the processes of circadian rhythm and sleep homeostatic.

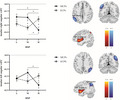

## 1 INTRODUCTION

Sleep–wake activity is regulated by two processes of circadian rhythm (C) and sleep homeostatic (S), which is known as the CS model.^1^ The process of circadian rhythm leads to rhythmic changes in sleep preferences within 24 h. During homeostatic process, homeostatic sleep pressure (HSP) accumulates with the increase of waking time and dissipates during sleep.^2^ Sleep homeostatic and circadian rhythm play an important role in mental health and even pathology.^3^, ^4^ Chronotype is a appropriate variable to investigate homeostatic sleep and circadian rhythm.^5^ Chronotype were usually divided into morningness, middle, and eveningness,^6^ which can be influenced by the processes of circadian rhythm and sleep homeostatic.^7^ Among them, differences in behavioral, physiological, and neurological performance between morningness chronotype (MCPs) and eveningness chronotype participants (ECPs) have been found. ^8^, ^9^, ^10^, ^11^, ^12^, ^13^, ^14^

Previous studies have found that chronotype is closely related to sleep homeostatic. The accumulation and dissipation of HSP in MCPs and ECPs are different, the former is faster in accumulation and dissipation than the latter.^7^, ^10^, ^15^, ^16^ HSP is associated with the diurnal neurophysiological activity in different chronotype. Peres et al. found that chronotype can significantly predict the peak of brain activation with the time‐of‐day changes on the bilateral supplementary motor area and the right rolandic operculum.^17^ Moreover, the different neurophysiological activity in chronotype can be affected by HSP. For instance, the activity of response inhibition‐related brain regions (e.g., the medial frontal gyrus and middle cingulate cortex) in MCPs significantly decreased from morning to evening, while ECPs remained stable or increased (e.g., the right inferior frontal gyrus and insula).^18^

Unlike the circadian rhythm, although we know that the level of slow wave activity decreased exponentially during sleep and increases gradually when awake,^19^ the neurophysiological basis of chronotype remained relatively unknown. Moreover, if the total sleep time of the previous day is limited, the HSP of the present day will increase.^20^ Therefore, the waking time of the day and the sleep duration of the previous day need to be considered to examine the effects of sleep pressure caused by sleep homeostatic.

Notably, studies about sleep homeostatic still encountered some problems to be resolved. First, using some fixed time points to measure HSP regardless of chronotype.^13^, ^18^ However, the same time point may have distinct meanings to physiological index and behavioral performance of different chronotype.^21^ Second, it is difficult to distinguish the separate effects between circadian rhythm and sleep homeostatic.^22^ The first problem can be solved by measuring the elapsed time awake.^23^ The solution to the second problem should focus on the control of rhythm differences within the same population through professional tools to distinguish chronotype, such as, the reduced version of Morningness‐Eveningness Questionnaire (rMEQ)^6^ and Munich Chronotype Questionnaire (MCTQ).^24^

Hodkinson et al. found that the functional connectivity and regional cerebral blood flow of inferior parietal lobule (IPL, including angular gyrus and middle temporal gyrus) decreased significantly as the waking time prolonged (morning vs. afternoon).^25^ Similarly, the amplitude of low‐frequency fluctuation of the angular gyrus in sleep deprivation group decreased significantly than the control group,^26^ which indicated that angular gyrus may be more sensitive to sleep homeostatic. Moreover, the insular was found to be sensitive to changes in HSP.^20^, ^23^ Some previous studies suggested that the insula is a critical brain area to maintain awareness, awakening and attention, and it is involved in the process of cognitive, affective, and regulatory functions.^27^, ^28^, ^29^, ^30^ The damage of the insular cause impairments to cognitive, affective, and regulatory functions.^30^ Interestingly, Sämann et al. have found that the resting‐state functional connectivity (rsFC) between left IPL (including left angular gyrus) and right insular decreased after partial sleep deprivation. The angular and insular as a sub‐region of the default mode network (DMN) and anti‐correlated network (ACN) of DMN, respectively,^20^ their rsFC changes may represent a key interface between externally and internally directed awareness. Also, the increased sleep pressure may affect individual's level of arousal and cognitive ability.^31^ Some previous studies suggested the increased rsFC between DMN and ACN was accompanied with increased cognitive performance,^27^ decreased fatigue degree,^29^ and increased vigilance level.^32^ However, some other studies found the increased rsFC was associated with higher levels of both hyperarousal and negative emotions.^33^ Taken together, the rsFC between the angular and insular may play an important role in the sleep homeostatic, and it may even be beneficial to predicting individual behavior performance and reveal its neural basis.

Based on the above‐mentioned research evidence, the present study focused on the rsFC between the insular and angular to reveal the diurnal neural activity basis with the increase of HSP. In addition, the current study also took the chronotype into account given the responses to HSP in MCPs can be different from ECPs. Specifically, we examined the neural mechanism underlying the interaction between chronotype and HSP with a large sample size. The participants were screened out by the rMEQ to determine their chronotype. All participants received rsfMRI scan at random time during the day. The HSP can be measured by the elapsed time awake,^23^ which is the difference between the scanning time and the waking time (D‐value). The greater D‐value means the higher HSP. In order to investigate the diurnal neural activity basis with the increase of HSP in different chronotype, independent component analysis was used to identify the components with insular and angular. We hypothesized that: (1) The rsFC between insular and angular will decreases as HSP increases regardless of chronotype; (2) MCPs and ECPs under the same HSP will show different rsFC pattern. Specifically, MCPs' rsFC between insular and angular may be lower than ECPs given their sleep pressure accumulates faster than ECPs.

## 2 METHOD

### 2.1 Participants

196 college students were selected by the scores of rMEQ (see the details in the Materials) from the Behavioral Brain Research Project of Chinese Personality at Southwest University,^34^ of which 4 participants were excluded due to fMRI data quality (head movement >2 mm or 2° in the scanner), including 64 MCPs (18.87 ± 0.95, female: 44) and 128 ECPs (18.72 ± 1.07, female: 86). There was no difference in age (*t* = 0.879, *p* = 0.381) and sex (*χ* ^2^ = 0.048, *p* = 0.827) between MCPs and ECPs. Participants with common physiological diseases, drugs and alcohol/tobacco dependence were excluded. Also, participants with abnormal sleep status were excluded. Of note, sleep quality in recent month and sleep status on the date of scanning were recorded.

### 2.2 Materials

The rMEQ measures individual sleep–wake timing preferences.^6^ It includes five questions with a total score range of 4–25. Participants were identified as ECPs with the scores lower than 11, as MCPs with their rMEQ scores higher than 18.

The MCTQ is another important measurement of chronotype by MSFsc (midpoint of sleep on free day without alarm clock).^24^ The MSFsc as a comparative parameter to verify the credibility of the rMEQ in identifying chronotype.

Pittsburgh Sleep Quality Index (PSQI) is an effective tool for measuring individual sleep quality.^35^ In our study, it was used to evaluate the sleep duration of the participants in the past month.

### 2.3 Protocol

All participants were randomly assigned to receive the rsfMRI scans at different time points during the day from 7:30 am to 22:30 pm. Participants were asked to maintain a regular sleep pattern as usual on the night before the scan. In order to investigate the response of each chronotype to HSP in an explicit way, we further divided HSP into three levels according to the characteristics of the awakening duration: low/medium/high (L/M/H) (Details are shown in Table S1).^21^

### 2.4 Acquisition of MRI data

All MR Images were acquired using a 3 T Siemens Prisma scanner with a standard head coil. High‐resolution T1‐weighted structural image was obtained using a three‐dimensional gradient sequence (repetition time (TR) = 2530 ms, time of echo (TE) = 2.98 ms, field of view (FOV) = 256 × 256 mm^2^, thickness = 1 mm, voxel size = 0.5 × 0.5 × 1 mm^3^, flip angle = 7°, resolution matrix = 512 × 512, slices = 192) and was used in subsequent resting‐state fMRI preprocessing. Then, the T2*‐weighted functional images were acquired with a gradient echo‐planar imaging (EPI) sequence (TR = 2000 ms, TE = 30 ms, FOV = 224 × 224 mm^2^, thickness = 2 mm, voxel size = 2 × 2 × 2 mm^3^, flip angle = 90°, acquisition matrix = 112 × 112, slices = 62).

### 2.5 Data preprocessing

All preprocessing steps were carried out using SPM12 (Wellcome Department of Cognitive Neurology, London, https://www.fil.ion.ucl.ac.uk/spm/software/spm12/) implemented in MATLAB 2019b. Functional images were (1) slice time corrected, (2) underwent motion correction and susceptibility artifact correction based on field map, (3) warped into Montreal Neurological Institute standard space using the diffeomorphic Anatomical Registration Through exponentiated Lie Algebra (Dartel) approach to realign the 3D anatomical data into Montreal Neurological Institute space,^36^ (4) smoothed spatially with a Gaussian kernel of 6 mm full width at half maximum (FWHM). Afterward, functional images further underwent denoising steps including (1) regression of the 12 realignment parameters of head motion artifacts, (2) regression of non‐relevant signals including breath, heartbeat, and other physiological noises and the remaining head movement artifacts using CompCor.^37^ In order to prevent the adverse effects of high motion participants on the removal of artifacts,^38^ 4 participants were excluded based on the follow‐up criteria: head movement >2 mm or 2°, average frame‐by‐frame displacement >0.2 mm or maximum frame‐by‐frame displacement >5 mm.^39^ In addition, outliers in the fMRI signals were monitored and removed by using an artifact detection tool (http://web.mit.edu/swg/software.htm). Specifically, if the scrubbing time lasted more than 1 min (30 TRs), participants' data will be excluded. Afterward, functional images underwent the regression of linear trend. Finally, functional images were filtered using a bandpass filter (0.008–0.09 Hz) to reduce the influence of low‐ and high‐frequency noise.

### 2.6 Insular‐angular rsFC calculation

Group Independent component analysis of the fMRI Toolbox (GIFT) was used to extract the resting‐state networks.^40^ The preprocessed data were imported into the GIFT software. First, a two‐step principal components analysis (PCA) was used to reduce subject‐specific data to 50 components, and the infomax algorithm applied for the data was repeated 20 times using ICASSO (http://www.cis.hutfi/projects/ica/icasso) to ensure the stability of the estimation.^41^ Of the 50 ICs, right insular, left and right angular were identified according to the AAL template (see Table 1). Finally, the rsFC between right insular and left angular and between right insular and right angular was extracted and Fisher's Z‐transformed by GIFT. The rsFC was calculated by the above method has many advantages.^42^

**TABLE 1:** Peak foci for right insular and angular defined by group independent component analysis (ICA)

Regions	Anat_label	MNI coordinates			*K*	*T*
		X	Y	Z		
Insular(IC 11)	Insular_R	44	18	−8	12,770	33.12
Angular(IC 13)	Angular_R	50	−62	40	2559	44.79
Angular(IC 21)	Angular_L	−46	−64	44	2455	42.00
	Temporal_Mid_R	62	−40	8	2442	16.45

*Note*: The threshold was set to *p* < 0.001, family wise error ‐corrected, with a cluster size >2000 voxels.

Abbreviations: Anat. label, anatomical labels from spm neuromorphometrics; IC, independent component; K, refers to cluster size; L, left; Mid, Middle; R, right.

### 2.7 Statistical analyses

To verify the reliability of rMEQ in screening out chronotype, the correlation between MSFsc and rMEQ score was calculated. Chi‐square test and *t*‐tests were used to analyze the differences of gender and age on MCPs and ECPs, respectively. The normal distribution tests of the data are shown in Table S2.

To evaluate whether there are sudden changes in participants' sleep–wake activity (i.e., sleep duration) which could influence the experimental effects, we used the first two items of the PSQI to assess routine habitual sleep and compare it with the corresponding items measured with the sleep diary (the date of the fMRI scanning).^43^ Hence, a 2 × 2 (chronotype [MCPs, ECPs] × sleeping habit [PSQI, Diary]) ANOVA was used.

To examine the different chronotype's response toward HSP revealed by rsFC between insula and angular (left and right), a 2 × 3 (chronotype [MCPs, ECPs] × HSP [L, M, H]) ANOVA was used. To control for multiple comparison issues with the significant level, we define the significant level as 0.05/2 and confidence intervals are 97.5%.

All of the above analyses were conducted using SPSS 22.

## 3 RESULTS

### 3.1 Demographic variables and questionnaire scores

There was no significant difference in sex (*χ* ^2^ = 0.048, *p* = 0.827) and age (*t* = 0.879, *p* = 0.381) between MCPs and ECPs (see Table 2). The MCPs showed poorer sleep quality compared with the ECPs as measured by the PSQI total score, *t* = −5.892, *p* < 0.001. (see Table 2).

**TABLE 2:** Description statistics of gender, age and PSQI on MCPs and ECPs

	MCPs (*N* = 64)	ECPs (*N* = 128)	*p*
Female, *N* (%)	68.75%	67.19%	0.827
Age	18.87 ± 0.95	18.72 ± 1.07	0.381
PSQI total scores	3.81 ± 2.03	5.97 ± 2.55	<0.001^***^

Abbreviations: ECPs, eveningness‐chronotype participants; MCPs, morningness‐chronotype participants; PSQI, Pittsburgh Sleep Quality Index.

***p < 0.001.

### 3.2 The check of sleeping habit

The results found that the main effect of chronotype (*F*[1190] = 0.413, *p* = 0.521, *η* ^2^ = 0.002) was insignificant. The main effect of sleeping habit (*F*[1190] = 10.309, *p* = 0.002, *η* ^2^ = 0.051) was insignificant, indicating the sleep duration of habitual night was longer than the night before scan. The chronotype × sleeping habit interaction effect was not significant (*F*[1190] = 0.026, *p* = 0.872, *ղ* ^2^ < 0.001), which indicated the night before scan and their habitual night was not different on MCPs or ECPs (see Table S3).

### 3.3 Validation of the rMEQ in identifying chronotype

**FIGURE 1:**
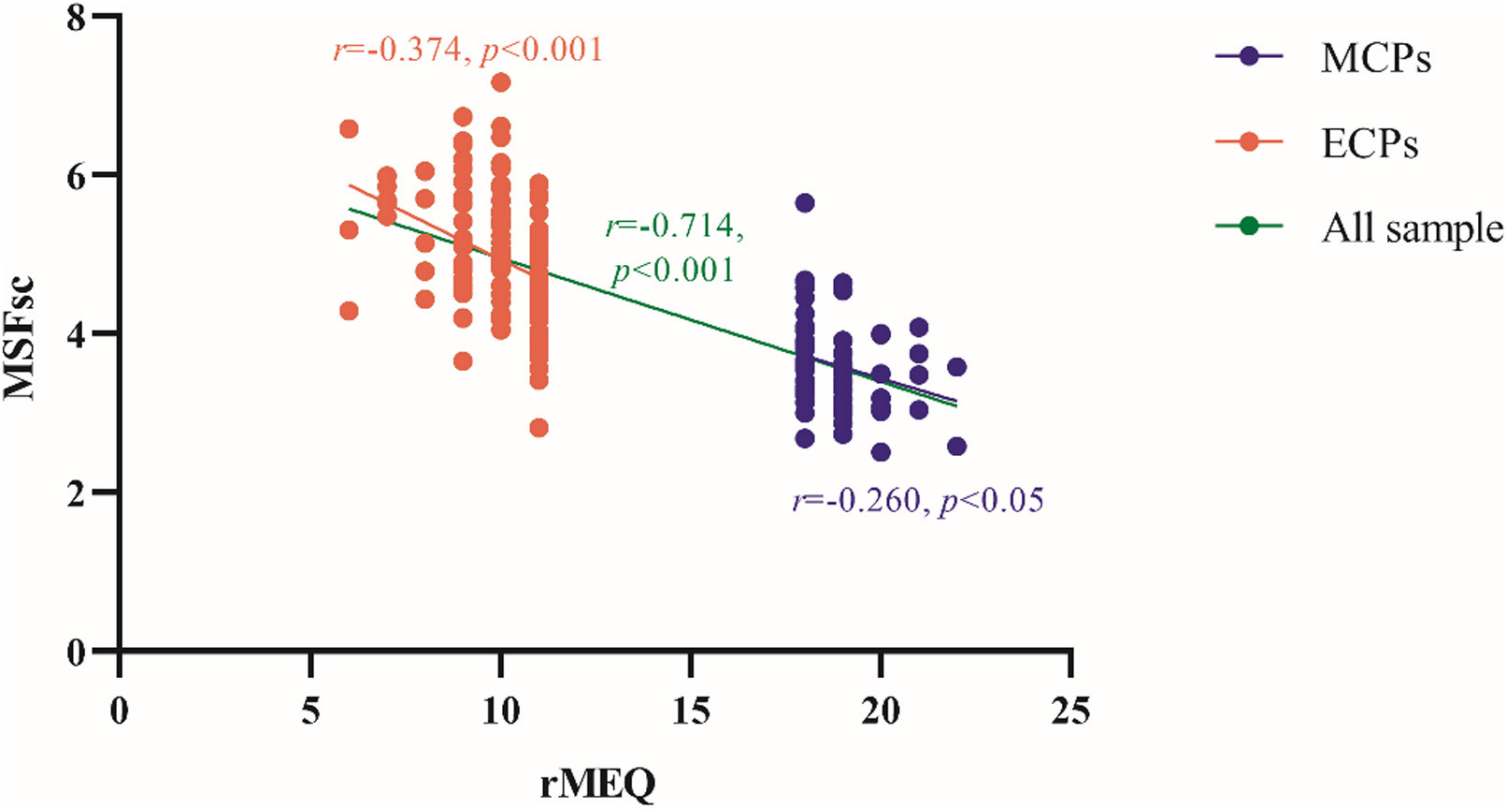
Correlation between rMEQ and MSFsc. MSFsc, midpoint of sleep on work free day without alarm clock; The lager rMEQ score indicates a preference for early chronotype; MCPs, morningness‐chronotype participants be screened by rMEQ; ECPs, eveningness‐chronotype participants be screened by rMEQ.

There is a significant negative correlation between rMEQ and MSFsc in the whole sample data (*r* = −0.714, *p* < 0.001, as well as in the MCPs group (*r* = −0.260, *p* < 0.05) and ECPs group (*r* = −0.374, *p* < 0.001) (also see Figure 1). These negative correlations indicated that the screening of the MCPs and ECPs through rMEQ have high representativeness and reliability.

### 3.4 Response of different chronotype to HSP revealed by insular‐right angular rsFC

**FIGURE 2:**
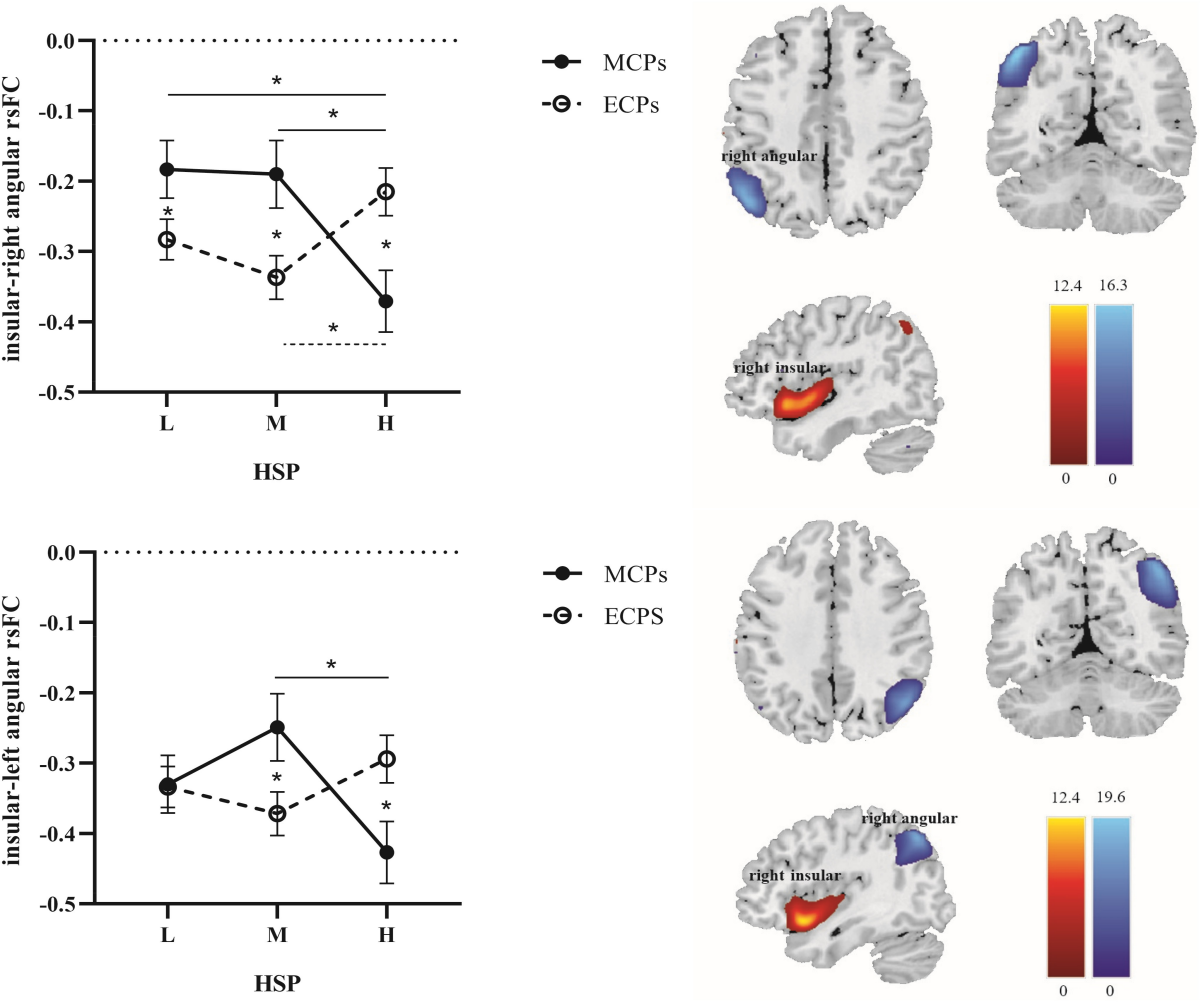
Resting‐state functional connectivity (rsFC) of different chronotype varies with the increased homeostatic sleep pressure (HSP). MCPs, morningness‐chronotype participants; ECPs, eveningness‐chronotype participants. L, Low HSP; M, Medium HSP; H, High HSP. *, *p* < 0.05.

For the negative rsFC between the right insular and right angular, the main effects of chronotype and HSP were not significant. The interaction between chronotype and HSP was significant, *F* (2,186) = 8.592, *p* < 0.001, *η* ^2^ = 0.085. Further post‐hoc analysis revealed significant differences in the rsFC of the ECPs (*F*(2,186) = 5.861, *p* = 0.003, *η* ^2^ = 0.059) and MCPs (*F*(2,186) = 3.491, *p* = 0.032, *η* ^2^ = 0.036) on different HSP conditions, respectively. Specifically, in MCPs, the rsFC under low and medium HSP level was significantly lower than high HSP level, but no significant difference between low and medium HSP levels. In ECPs, the rsFC under medium HSP level was significantly higher than high HSP level, but no significant difference between the other levels (Figures 2 and S1, Tables S4 and S5).

### 3.5 Response of different chronotype to HSP revealed by insular‐left angular rsFC

For negative rsFC between the right insular and left angular, the main effects of chronotype and HSP were not significant. The interaction between chronotype and HSP was significant *F*(2, 186) = 5.117, *p* = 0.007, *η* ^2^ = 0.052. Further post hoc analysis found the rsFC at high HSP level was significantly higher than medium HSP level in MCPs (*F*(2,186) = 3.753, *p* = 0.025, *η* ^2^ = 0.039). The rsFC in ECPs tended to decrease with the increase of HSP; however, it did not reach a significant level (*F*(2,186) = 1.415, *p* = 0.246, *η* ^2^ = 0.015) (Figures 2 and S2, Tables S4 and S5).

### 3.6 Differences between chronotype under the same HSP

The rsFC between right insular and right angular in MCPs was significantly lower than that in ECPs when HSP is low (*F*[1186] = 4.024, *p* = 0.046, *η* ^2^ = 0.021) and medium (*F*[1186] = 6.648, *p* = 0.011, *η* ^2^ = 0.035). While the opposite pattern was found when HSP is high, namely, the rsFC in MCPs was higher than that in ECPs (*F*[1186] = 7.760, *p* = 0.006, *η* ^2^ = 0.040) (Figures 2 and S1, Tables S4 and S5).

On the rsFC between right insular and left angular, there was not difference of chronotype (*F*[1186] = 0.008, *p* = 0.929, *η* ^2^ < 0.001) when HSP is low. However, when HSP is medium, the rsFC of MCPs was significantly lower than that in ECPs (*F*[1186] = 4.603, *p* = 0.033, *η* ^2^ < 0.024), while the opposite pattern was found when HSP is high (*F*[1186] = 5.632, *p* = 0.019, *η* ^2^ < 0.029), namely, the rsFC in MCPs was significantly higher than that in the ECPs (Figures 2 and S1, Tables S4 and S5).

## 4 DISCUSSION

The current study presented distinct neural response of different chronotype to sleep pressure revealed by rsFC between insula and bilateral angular for the first time. The negative rsFC between right insular and bilateral angular increased in MCPs while decreased in ECPs with the accumulation of sleep pressure. In addition, this study revealed that different chronotype exhibited different rsFC pattern under the same sleep pressure. All together, these findings provided neurobiological evidence of sleep pressure affecting the chronotype.

### 4.1 Distinct rsFC change pattern between ECPs and MCPs in response to the accumulation of sleep pressure

Morningness chronotype responded very differently from ECPs on rsFC between insula and angular as diurnal sleep pressure accumulates. The negative rsFC between right insula and angular was decreased in ECPs under high sleep pressure compared with that under low and medium sleep pressure. Bilateral angular gyrus are important regions of the default mode network (DMN) and insula is a hub region of anti‐correlated network (ACN). Previous study indicated that increased sleep pressure led to a decline in negative rsFC between DMN and CAN,^20^ which is consistent with our findings on ECPs. DMN and ACN were considered as key brain networks connecting internal and external consciousness. The rsFC between DMN and ACN diminished with increased sleep pressure, which further led to increased sleepiness, difficulty sustained attention, decreased cognitive performance, etc.^20^ Previous studies also found that the negative rsFC of the DMN and ACN was associated with cognition,^27^ fatigue,^29^ and vigilance.^32^ And the stronger anti‐correlation is accompanied with the better performance. These studies implicitly suggested that the results of ECPs' performance may decrease with increased diurnal sleep pressure. However, the negative rsFC between right insular and left angular did not show corresponding decrease with the increase of sleep pressure in ECPs. This inconsistency may be due to the different sensitivity to sleep pressure between left and right angular, the right angular gyrus encountered higher activity after sleep deprivation compared with the left angular gyrus.^44^

On the contrary, the negative rsFC between right insular and right angular increased with the HSP in MCPs, which is inconsistent with a previous study.^20^ The rsFC of the MCPs gradually increased with the accumulation of sleep pressure. This may indicate that hyperarousal occurred in the MCPs. Shi et al. pointed out the enhanced DMN‐ACN functional connectivity was involved in sustained hypervigilance and hyperarousal, and it could reflect the function of self‐reflection, emotional regulation and cognitive control. Also, the cortisol secretion levels of MCPs were significantly higher than ECPs at 10 h after awakening,^45^ which may be manifested by heightened arousal, including hypersensivity to various stimuli and/or hypervigilance.^46^ Moreover, MCPs showed a higher total amount of cortisol and there was a significantly shortened phase of cortisol increase within the first hour after awakening,^47^ which suggested that MCPs may increase cortisol secretion levels after a longer period of awakening and enhance arousal. Although stronger anti‐correlation of rsFC between DMN and ACN may be accompanied by better performance, it may also represent mere negative emotion and arousal.^33^ Nevertheless, whether hyperarousal occurred in the MCPs still needed to be examined in future studies.

In addition, we found the negative rsFC between right insular and left angular is higher on high than medium sleep pressure level in MCPs while no difference was found between high and low sleep pressure level. These change pattern of rsFC may be due to the different sensitivity of the angular to sleep pressure as previous research has found.^20^, ^44^ Taken together, these results not only provided evidence for the validation and in‐depth understanding of CS models but also provided the possibility of developing optimal activity plans based on individuals' sleep pressure change patterns.

### 4.2 The different neural response between ECPs and MCPs under the same sleep pressure level

When sleep pressure remained at a certain level, MCPs, and ECPs responded differently in the rsFC. Under low sleep pressure, the right insular‐right angular negative rsFC of ECPs was larger than that of MCPs, which could be explained by the difference in cortisol secretion levels between ECPs and MCPs. A previous studies pointed out there was a significantly prolonged phase of cortisol increase within the first hour after awakening in ECPs.^47^ However, there was no significant difference in terms of the right insular‐left angular rsFC between ECPs and MCPs. This may be due to the lack of sensitivity of ECPs to sleep pressure,^44^ as we found that right insular‐left angular rsFC did not change with the increase in HSP.

Under medium sleep pressure, MCPs had stronger negative rsFC between the right insular‐bilateral angular than ECPs. MCPs has a faster accumulation and dissipation speed of diurnal HSP,^7^, ^10^, ^15^, ^16^ which may resulted in greater sleep pressure. Some previous studies found the stronger anti‐correlation rsFC of DMN and ACN was associated with better behavioral performance,^27^, ^32^ which may suggested ECPs had a better performance under low and medium sleep pressure than MCPs.

Under high sleep pressure, the pattern was reversed, the right insular‐bilateral angular negative rsFC was smaller in MCPs than that in ECPs. As we stated previously, MCPs may had hyperarousal under high sleep pressure.^33^ However, the neural mechanism of the difference between the two chronotype at high sleep pressure still needs to be studied further, especially the examination about whether hyperarousal occurred in MCPs under high sleep pressure.

### 4.3 Research implications and future directions

Despite the number of strengths of this innovative study, the current study contained several limitations too. First, this study used a between‐subject design, so the experimental effects are reliable on the strict control of the individual differences. By doing so, sleep duration, gender, and age were matched between groups in the study. Second, the effect of the circadian rhythm was not completely eliminated after controlling other additional variables (a polynomial curve fitting for diurnal rsFC, see Figure S2), which suggests that we need to take effective methods to separate the effects of circadian rhythm and sleep homeostatic in further studies. Third, the sleep debt due to the sleep–wake activity between work day and free day was not considered in our research. However, sleep duration measured on the night before the scan and that measured by habitual sleep did not differ between MCPs and ECPs (see Table S3). This indicated the impact of sleep debt does not affect the results. Finally, the current study did not include the intermediate chronotype participants; hence, our results may be limited. However, previous research has found extreme chronotype are more vulnerable on sleep health,^48^ emotion,^49^ and cognition.^13^

## 5 CONCLUSION

This study found the rsFC between insula and angular changed significantly with the diurnal sleep pressure accumulation, and the change patterns were quite different between MCPs and ECPs. Moreover, the same HSP has different meanings for MCPs and ECPs. These findings may be helpful in the understanding of the individual difference in the level of alertness, cognitive performance, emotional state, etc. The different change modes of insular‐angular rsFC between MCPs and ECPs may provide neuroimaging indicators to distinguishing them under diurnal sleep pressure. The present study adds to the understanding of the processes of circadian rhythm and sleep homeostatic.

## CONFLICTS OF INTEREST

The authors declare that they have no conflicts of interest.

## Supporting information


Appendix S1

## Data Availability

The fMRI data sharing is not applicable to this article, because the project of the fMRI data source of this article is still in progress and it is held jointly by the corresponding author and the other project leaders. Before the data for the entire project is made public, an agreement with the corresponding author and other project leaders is required if it is necessary to obtain the fMRI data. Data of other variables are available upon request.

## References

[1] Borbely AA , Daan S , Wirz‐Justice A , Deboer T . The two‐process model of sleep regulation: a reappraisal. J Sleep Res. 2016;25(2):131‐143. 26762182 10.1111/jsr.12371

[2] Robillard R , Prince F , Boissonneault M , Filipini D , Carrier J . Effects of increased homeostatic sleep pressure on postural control and their modulation by attentional resources. Clin Neurophysiol. 2011;122(9):1771‐1778. 21396885 10.1016/j.clinph.2011.02.010

[3] Taylor BJ , Hasler BP . Chronotype and mental health: recent advances. Curr Psychiat Rep. 2018;20(8):59. 10.1007/s11920-018-0925-8 30039327

[4] Kivelä L , Papadopoulos MR , Antypa N . Chronotype and psychiatric disorders. Curr Sleep Med Rep. 2018;4(2):94‐103. 29888167 10.1007/s40675-018-0113-8 PMC5972175

[5] Gaggioni G , Maquet P , Schmidt C , Dijk DJ , Vandewalle G . Neuroimaging, cognition, light and circadian rhythms. Front Syst Neurosci. 2014;8:126. 25071478 10.3389/fnsys.2014.00126 PMC4086398

[6] Adan A , Almirall H . Horne & Östberg morningness‐eveningness questionnaire: a reduced scale. Personal Individ Differ. 1991;12(3):241‐253.

[7] Mongrain V , Carrier J , Dumont M . Circadian and homeostatic sleep regulation in morningness‐eveningness. J Sleep Res. 2006;15(2):162‐166. 16704571 10.1111/j.1365-2869.2006.00532.x

[8] Kerkhof GA , VanDongen HPA . Morning‐type and evening‐type individuals differ in the phase position of their endogenous circadian oscillator. Neurosci Lett. 1996;218(3):153‐156. 8945751 10.1016/s0304-3940(96)13140-2

[9] Mongrain V , Lavoie S , Selmaoui B , Paquet J , Dumont M . Phase relationships between sleep‐wake cycle and underlying circadian rhythms in morningness‐eveningness. J Biol Rhythms. 2004;19(3):248‐257. 15155011 10.1177/0748730404264365

[10] Taillard J , Philip P , Coste O , Sagaspe P , Bioulac B . The circadian and homeostatic modulation of sleep pressure during wakefulness differs between morning and evening chronotypes. J Sleep Res. 2003;12(4):275‐282. 14633238 10.1046/j.0962-1105.2003.00369.x

[11] Vitale JA , Roveda E , Montaruli A , et al. Chronotype influences activity circadian rhythm and sleep: differences in sleep quality between weekdays and weekend. Chronobiol Int. 2015;32(3):405‐415. 25469597 10.3109/07420528.2014.986273

[12] Facer‐Childs E , Brandstaetter R . The impact of circadian phenotype and time since awakening on diurnal performance in athletes. Curr Biol. 2015;25(4):518‐522. 25639241 10.1016/j.cub.2014.12.036

[13] Facer‐Childs E , Campos BM , Middleton B , Skene DJ , Bagshaw AP . Circadian phenotype impacts the brain's resting‐state functional connectivity, attentional performance, and sleepiness. Sleep. 2019;42(5):zsz033. 30763951 10.1093/sleep/zsz033 PMC6519915

[14] Blautzik J , Vetter C , Peres I , et al. Classifying fMRI‐derived resting‐state connectivity patterns according to their daily rhythmicity. Neuroimage. 2013;71:298‐306. 22906784 10.1016/j.neuroimage.2012.08.010

[15] Mongrain V , Carrier J , Dumont M . Chronotype and sex effects on sleep architecture and quantitative sleep EEG in healthy young adults. Sleep. 2005;28(7):819‐827. 16124660 10.1093/sleep/28.7.819

[16] Schmidt C , Collette F , Leclercq Y , et al. Homeostatic sleep pressure and responses to sustained attention in the suprachiasmatic area. Science. 2009;324(5926):516‐519. 19390047 10.1126/science.1167337

[17] Peres I , Vetter C , Blautzik J , et al. Chronotype predicts activity patterns in the neural underpinnings of the motor system during the day. Chronobiol Int. 2011;28(10):883‐889. 22080733 10.3109/07420528.2011.619084

[18] Song JJ , Feng P , Zhao XY , et al. Chronotype regulates the neural basis of response inhibition during the daytime. Chronobiol Int. 2018;35(2):208‐218. 29144173 10.1080/07420528.2017.1392550

[19] Borbély AA . A two process model of sleep regulation. Hum Neurobiol. 1982;1(3):195‐204. 7185792

[20] Sämann PG , Tully C , Spoormaker VI , et al. Increased sleep pressure reduces resting state functional connectivity. Magn Reson Mater Phy. 2010;23(5–6):375‐389. 10.1007/s10334-010-0213-z 20473549

[21] Taillard J , Philip P , Claustrat B , et al. Time course of neurobehavioral alertness during extended wakefulness in morning‐ and evening‐type healthy sleepers. Chronobiol Int. 2011;28(6):520‐527. 21797780 10.3109/07420528.2011.590623

[22] Facer‐Childs E , de Campos BM , Middleton B , Skene DJ , Bagshaw AP . Temporal organisation of the brain's intrinsic motor network: the relationship with circadian phenotype and motor performance. Neuroimage. 2021;232:117840. 33577933 10.1016/j.neuroimage.2021.117840 PMC8214225

[23] Muto V , Jaspar M , Meyer C , et al. Local modulation of human brain responses by circadian rhythmicity and sleep debt. Science. 2016;353(6300):687‐690. 27516598 10.1126/science.aad2993

[24] Roenneberg T , Wirz‐Justice A , Merrow M . Life between clocks: daily temporal patterns of human chronotypes. J Biol Rhythms. 2003;18(1):80‐90. 12568247 10.1177/0748730402239679

[25] Hodkinson DJ , O'Daly O , Zunszain PA , et al. Circadian and homeostatic modulation of functional connectivity and regional cerebral blood flow in humans under normal entrained conditions. J Cerebr Blood F Met. 2014;34(9):1493‐1499. 10.1038/jcbfm.2014.109 PMC4158665 24938404

[26] Dai XJ , Liu CL , Zhou RL , et al. Long‐term total sleep deprivation decreases the default spontaneous activity and connectivity pattern in healthy male subjects: a resting‐state fMRI study. Neuropsych Dis Treat. 2015;11:761‐772. 10.2147/NDT.S78335 PMC4372006 25834451

[27] Chen HY , Li YX , Liu Q , et al. Abnormal interactions of the salience network, central executive network, and default‐mode network in patients with different cognitive impairment loads caused by Leukoaraiosis. Front Neural Circuit. 2019;13:42. 10.3389/fncir.2019.00042 PMC6592158 31275116

[28] Medford N , Critchley HD . Conjoint activity of anterior insular and anterior cingulate cortex: awareness and response. Brain Struct Funct. 2010;214(5–6):535‐549. 20512367 10.1007/s00429-010-0265-x PMC2886906

[29] Zhao QH , Li H , Yu XY , et al. Abnormal resting‐state functional connectivity of insular subregions and disrupted correlation with working memory in adults with attention deficit/hyperactivity disorder. Front Psychiatry. 2017;8:200. 29075206 10.3389/fpsyt.2017.00200 PMC5641567

[30] Ibanez A , Gleichgerrcht E , Manes F . Clinical effects of insular damage in humans. Brain Struct Funct. 2010;214(5–6):397‐410. 20512375 10.1007/s00429-010-0256-y

[31] Czisch M , Wehrle R , Harsay H , et al. On the need of objective vigilance monitoring: effects of sleep loss on target detection and task‐negative activity using combined EEG/fMRI. Front Neurol. 2012;3:67. 22557992 10.3389/fneur.2012.00067 PMC3338067

[32] Sripada RK , King AP , Welsh RC , et al. Neural dysregulation in posttraumatic stress disorder: evidence for disrupted equilibrium between salience and default mode brain networks. Psychosom Med. 2012;74(9):904‐911. 23115342 10.1097/PSY.0b013e318273bf33 PMC3498527

[33] Shi L , Sun J , Wu X , et al. Brain networks of happiness: dynamic functional connectivity among the default, cognitive and salience networks relates to subjective well‐being. Soc Cogn Affect Neurosci. 2018;13(8):851‐862. 30016499 10.1093/scan/nsy059 PMC6123521

[34] Yang S , Tian Y , He Q , Feng T , Chen H , Lei X . Enhanced anti‐correlation between the dorsal attention and default‐mode networks: a resting‐state fMRI study of acute insomnia. Neuroscience. 2021;467:47‐55. 34022324 10.1016/j.neuroscience.2021.05.014

[35] Buysse DJ , Reynolds CF , Monk TH , Berman SR , Kupfer DJ . The Pittsburgh sleep quality index ‐ a new instrument for psychiatric practice and research. Psychiatry Res. 1989;28(2):193‐213. 2748771 10.1016/0165-1781(89)90047-4

[36] Ashburner J . A fast diffeomorphic image registration algorithm. Neuroimage. 2007;38(1):95‐113. 17761438 10.1016/j.neuroimage.2007.07.007

[37] Muschelli J , Nebel MB , Caffo BS , Barber AD , Pekar JJ , Mostofsky SH . Reduction of motion‐related artifacts in resting state fMRI using aCompCor. Neuroimage. 2014;96:22‐35. 24657780 10.1016/j.neuroimage.2014.03.028 PMC4043948

[38] Parkes L , Fulcher B , Yucel M , Fornito A . An evaluation of the efficacy, reliability, and sensitivity of motion correction strategies for resting‐state functional MRI. Neuroimage. 2018;171:415‐436. 29278773 10.1016/j.neuroimage.2017.12.073

[39] Power JD , Plitt M , Laumann TO , Martin A . Sources and implications of whole‐brain fMRI signals in humans. Neuroimage. 2017;146:609‐625. 27751941 10.1016/j.neuroimage.2016.09.038 PMC5321814

[40] Calhoun VD , Adali T . Multisubject independent component analysis of fMRI: a decade of intrinsic networks, default mode, and neurodiagnostic discovery. IEEE Rev Biomed Eng. 2012;5:60‐73. 23231989 10.1109/RBME.2012.2211076 PMC4433055

[41] Himberg J , Hyvarinen A , Esposito F . Validating the independent components of neuroimaging time series via clustering and visualization. Neuroimage. 2004;22(3):1214‐1222. 15219593 10.1016/j.neuroimage.2004.03.027

[42] Calhoun VD , de Lacy N . Ten key observations on the analysis of resting‐state functional MR imaging data using independent component analysis. Neuroimaging Clin N Am. 2017;27(4):561‐579. 28985929 10.1016/j.nic.2017.06.012 PMC5657522

[43] van Hees VT , Sabia S , Anderson KN , et al. A novel, open access method to assess sleep duration using a wrist‐worn accelerometer. Plos One. 2015;10(11):e0142533. 26569414 10.1371/journal.pone.0142533 PMC4646630

[44] Nechifor RE , Ciobanu D , Vonica CL , et al. Social jetlag and sleep deprivation are associated with altered activity in the reward‐related brain areas: an exploratory resting‐state fMRI study. Sleep Med. 2020;72:12‐19. 32540632 10.1016/j.sleep.2020.03.018

[45] Oginska H , Fafrowicz M , Golonka K , Marek T , Mojsa‐Kaja J , Tucholska K . Chronotype, sleep loss, and diurnal pattern of salivary cortisol in a simulated daylong driving. Chronobiol Int. 2010;27(5):959‐974. 20636209 10.3109/07420528.2010.489412

[46] Chakarov S , Petkova R , Russev G , Zhelev N . DNA damage and the circadian clock. Biodiscovery. 2014;13:e8960.

[47] Abbruzzese E , Klingmann A , Ehlert U . Chronotype and cortisol awakening response (CAR). The influence of the chronotype on the awakening response of cortisol in the morning. Adv Soc Sci Res J. 2014;1(7):115‐121.

[48] Nunez P , Perillan C , Arguelles J , Diaz E . Comparison of sleep and chronotype between senior and undergraduate university students. Chronobiol Int. 2019;36(12):1626‐1637. 31500452 10.1080/07420528.2019.1660359

[49] Horne CM , Norbury R . Late chronotype is associated with enhanced amygdala reactivity and reduced fronto‐limbic functional connectivity to fearful versus happy facial expressions. Neuroimage. 2018;171:355‐363. 29339309 10.1016/j.neuroimage.2018.01.025

