# Supplementary material for: Distinct neural responses of morningness and eveningness chronotype to homeostatic sleep pressure revealed by resting‐state functional magnetic resonance imaging
Source: CNS Neurosci Ther. 2022 Jun 14;28(9):1439–46. doi: 10.1111/cns.13887 (PMC9344083; doi:10.1111/cns.13887)
Supplement: Supplementary file 1 — Appendix S1 [file CNS-28-1439-s001.doc]

**Table S1. Grouping according to sleep homeostatic pressure**

| HSP | D-value（h） | MCPs (n) | ECPs (n) | *χ*^2^ | *p* |
| --- | --- | --- | --- | --- | --- |
| L | <6 | 25 | 50 | 6.00 | 0.199 |
| M | 6-11 | 18 | 43 |
| H | >11 | 21 | 35 |

MCPs, morningness-chronotype participants; ECPs, eveningness-chronotype participants. HSP: homeostatic sleep pressure, L, Low HSP; M, Medium HSP; H, High HSP. D-value, the difference between scanning time and waking time.

**Table S2. Testing of normal distribution (****Kolmogorov-Smirnov Test) on chronotype (rMEQ, MSFsc) and sleep duration (PSQI, dairy).**

|  | Samples | Mean | SD | *p* |
| --- | --- | --- | --- | --- |
| rMEQ | All (192) | 12.917 | 4.380 | .000 |
|  | MCPs (64) | 18.875 | 1.062 | .000 |
|  | ECPs (128) | 9.938 | 1.209 | .000 |
| MSFsc(MCTQ) | All (192) | 4.502 | .963 | .200 |
|  | MCPs (64) | 3.600 | .582 | .200 |
|  | ECPs (128) | 4.953 | .782 | .200 |
| SD_PSQI | All (192) | 7.167 | 1.194 | .040 |
|  | MCPs (64) | 7.091 | 1.145 | .054 |
|  | ECPs (128) | 7.205 | 1.221 | .055 |
| SD_dairy | All (192) | 7.214 | 1.190 | .000 |
|  | MCPs (64) | 7.134 | 1.145 | .056 |
|  | ECPs (128) | 7.254 | 1.215 | .028 |

MCPs, morningness-chronotype participants; ECPs, eveningness-chronotype participants. *p*>0.05, normal distribution. From this table, all sample of rMEQ is not normal distribution, we used Spearman's correlation to analyze the relationship between rMEQ and MSFsc.

**Table S3. The descriptive statistics of sleep duration on MCPs and ECPs.**

|  | MCPs(M±SD) | ECPs(M±SD) |
| --- | --- | --- |
| sleep duration (PSQI) | 7:05 ± 69’ | 7:13 ± 73’ |
| sleep duration (Diary) | 7:08 ± 69’ | 7:15 ± 73’ |

MCPs, morningness-chronotype participants; ECPs, eveningness-chronotype participants. PSQI, Pittsburgh Sleep Quality Index. Diary, sleep diary.

**Table S4. The descriptive statistics of rsFC on MCPs and ECPs.**

|  |  | MCPs(M±SD) | | ECPs(M±SD) | |
| --- | --- | --- | --- | --- | --- |
|  | HSP | Mean | SD | Mean | SD |
| Insular-right angular | L | -.183 | .198 | -.283 | .194 |
|  | M | -.190 | .244 | -.337 | .184 |
|  | H | -.371 | .203 | -.215 | .215 |
| Insular-left angular | L | -.330 | .200 | -.334 | .228 |
|  | M | -.249 | .187 | -.372 | .181 |
|  | H | -.427 | .193 | -.294 | .208 |

MCPs, morningness-chronotype participants; ECPs, eveningness-chronotype participants. HSP: homeostatic sleep pressure, L, Low HSP; M, Medium HSP; H, High HSP.

**Table S5. The two-way ANOVA for rsFC between chronotype and HSP.**

|  | Factor | *dfs* | *F* | *p* | *η²* |
| --- | --- | --- | --- | --- | --- |
| Insular-right angular | Chronotype | 1,186 | .926 | .337 | .005 |
|  | HSP | 2,186 | 1.286 | .279 | .014 |
|  | Chronotype × HSP | 2,186 | 8.592 | <.001*** | .085 |
| Insular-left angular | Chronotype | 1,186 | .004 | .947 | .000 |
|  | HSP | 2,186 | .790 | .455 | .008 |
|  | Chronotype × HSP | 2,186 | 5.117 | <.01** | .052 |

Chronotype, including MCPs (morningness-chronotype participants) and ECPs (eveningness-chronotype participants). HSP: homeostatic sleep pressure, L, Low HSP; M, Medium HSP; H, High HSP. *, p<0.05; **, p<0.01; ***, p<0.001.


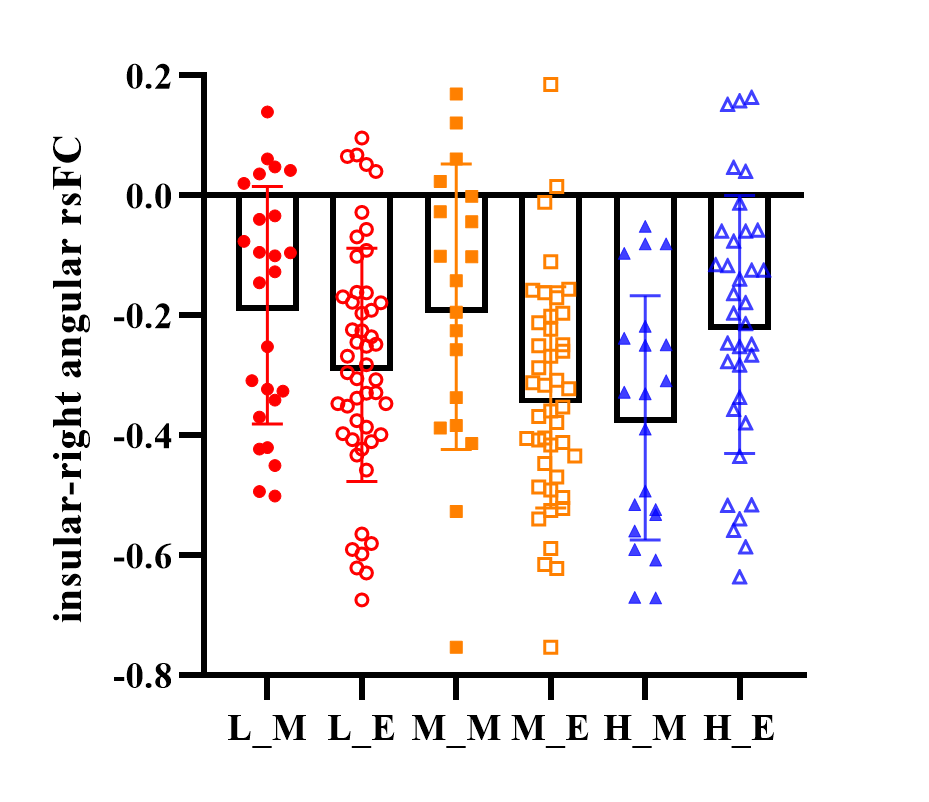

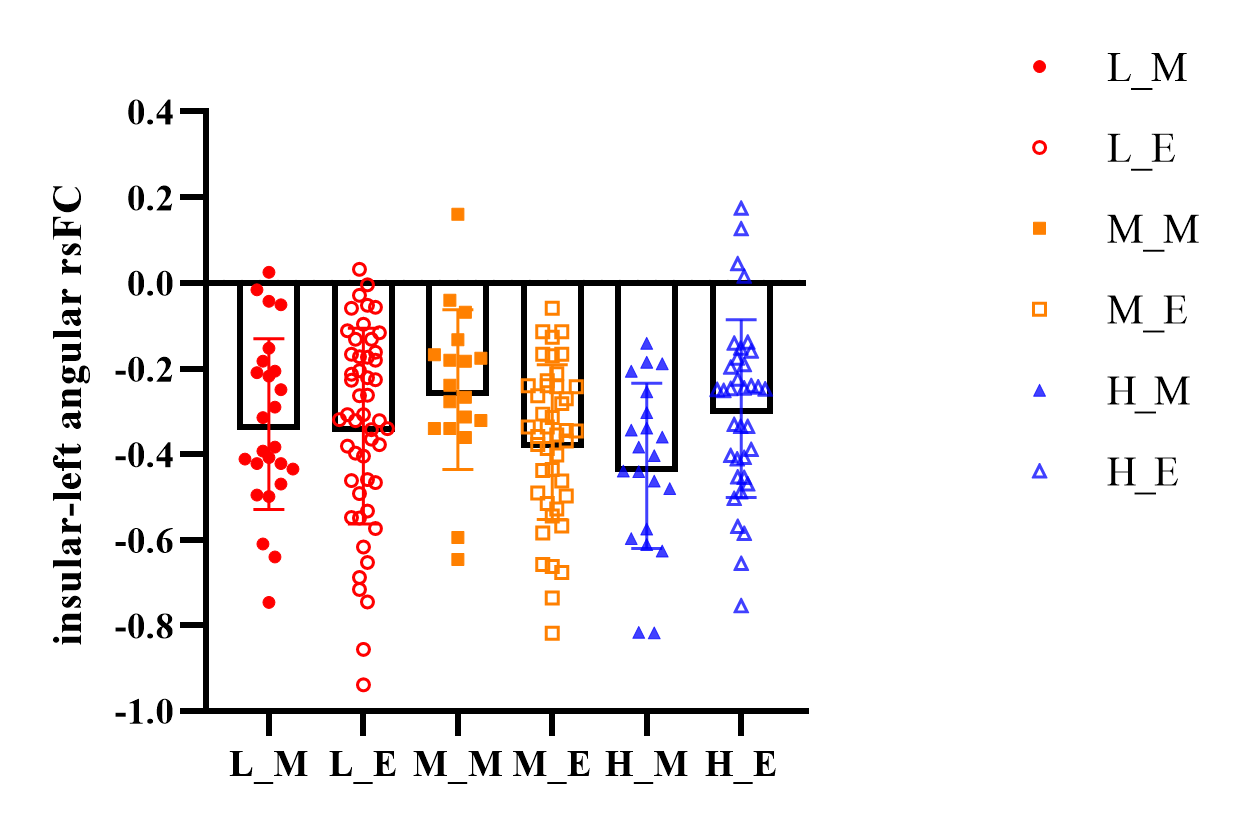


**Figure S1.** The resting state functional connectivity (rsFC) of different chronotype (M, morningness-chronotype participants; E, eveningness-chronotype participants) on homeostatic sleep pressure (L, Low HSP; M, Medium HSP; H, High HSP.).


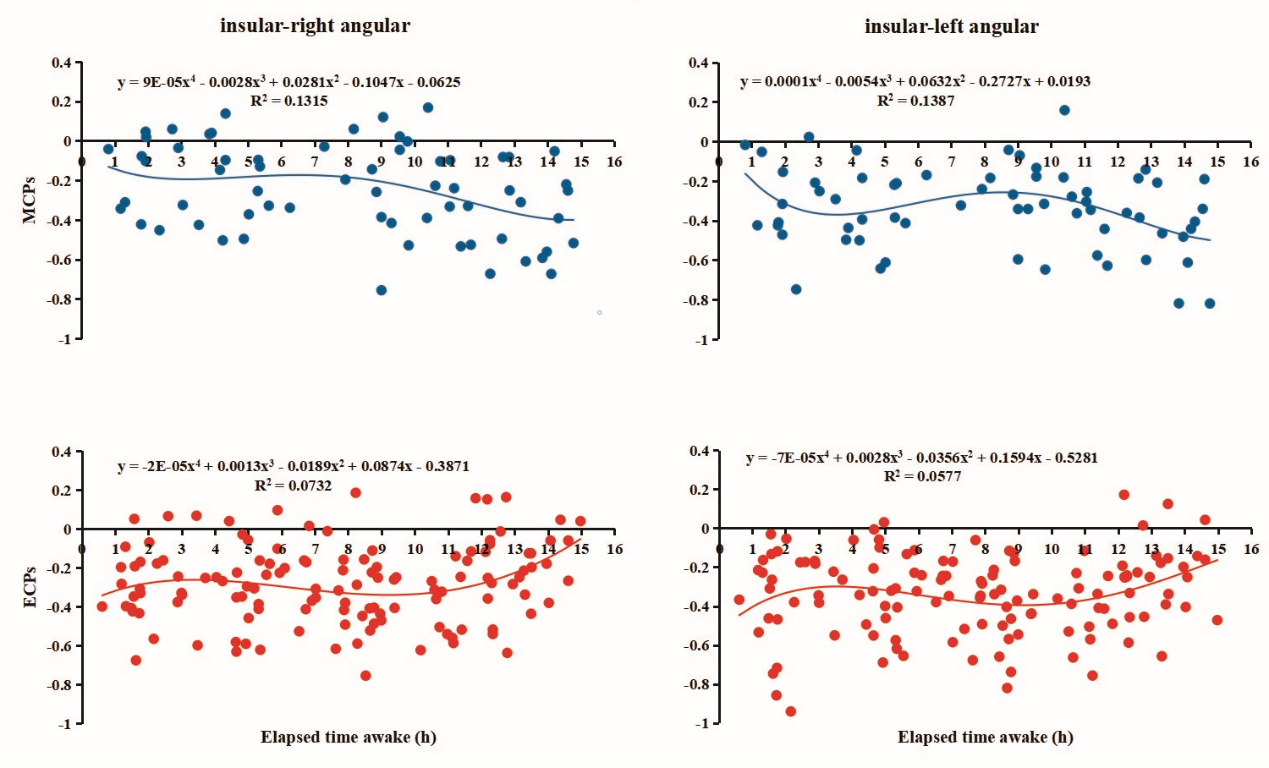


**Figure S2.** The rhythmic fluctuations of rsFC of right insular-angular on MCPs and ECPs. rsFC, resting state functional connectivity. MCPs, morningness-chronotype participants; ECPs, eveningness-chronotype participants.


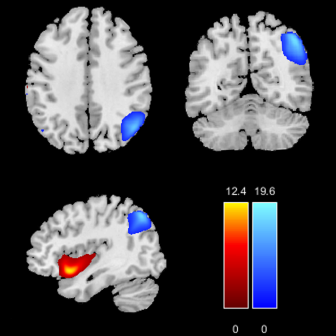

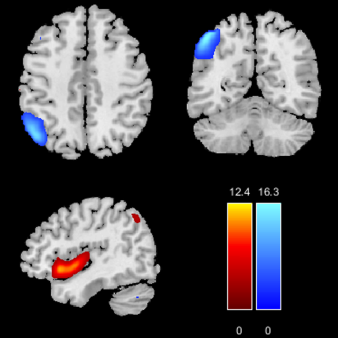


Figure R1. The Original activation imageon Insular and Angular.


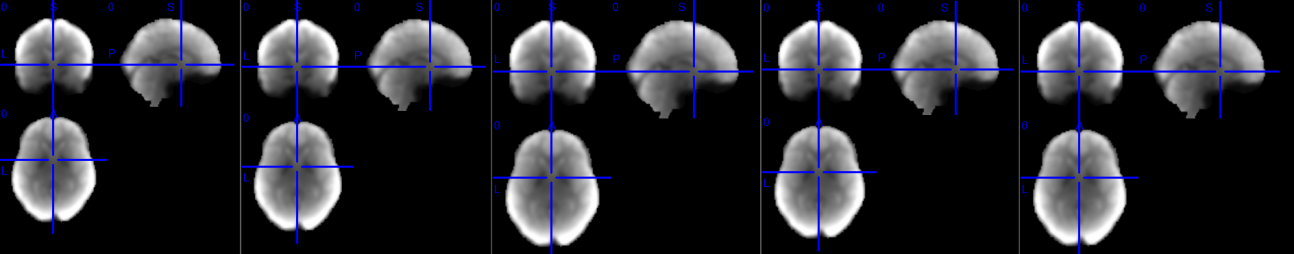

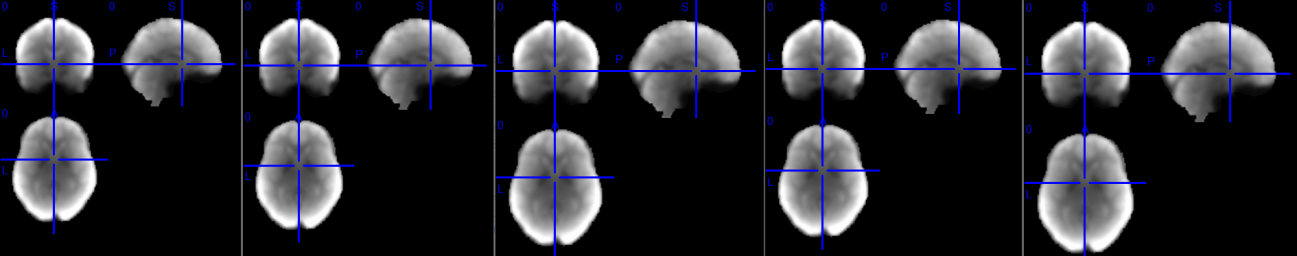


Figure R2. The first 10 volumes of our data from one participant, note that this data has been preprocessed.

Table R1. The description and statistics of demographic information.

|  | | MCPs_L | MCPs_M | MCPs_H | ECPs_L | ECPs_M | ECPs_H | F **/** *χ*^2^ | *p* |
| --- | --- | --- | --- | --- | --- | --- | --- | --- | --- |
| Age (M±SD) | | 18.716±0.890 | 18.928±0.802 | 18.995±1.140 | 18.714±0.970 | 18.777±1.354 | 18.689±0.820 | .364 | .873 |
| Gender (n) | female | 19 | 13 | 12 | 31 | 27 | 28 | 5.665 | .340 |
|  | male | 6 | 5 | 9 | 19 | 16 | 7 |  |  |

MCPs, morningness-chronotype participants; ECPs, eveningness-chronotype participants. HSP: homeostatic sleep pressure, L, Low HSP; M, Medium HSP; H, High HSP. D-value, the difference between scanning time and waking time.
